# Differential gene expression analysis of symbiotic and aposymbiotic *Exaiptasia* anemones under immune challenge with *Vibrio coralliilyticus*

**DOI:** 10.1002/ece3.5403

**Published:** 2019-06-30

**Authors:** Charles L. Roesel, Steven V. Vollmer

**Affiliations:** ^1^ Marine Science Center Northeastern University Nahant Massachusetts USA

**Keywords:** aposymbiotic, Cnidarian, *Exaiptasia*, gene expression, immune, symbiotic, transcriptome

## Abstract

Anthozoans are a class of Cnidarians that includes scleractinian corals, anemones, and their relatives. Despite a global rise in disease epizootics impacting scleractinian corals, little is known about the immune response of this key group of invertebrates. To better characterize the anthozoan immune response, we used the model anemone *Exaiptasia pallida* to explore the genetic links between the anthozoan–algal symbioses and immunity in a two‐factor RNA‐Seq experiment using both symbiotic and aposymbiotic (menthol‐bleached) *Exaiptasia pallida* exposed to the bacterial pathogen *Vibrio coralliilyticus*. Multivariate and univariate analyses of *Exaiptasia* gene expression demonstrated that exposure to live *Vibrio coralliilyticus* had strong and significant impacts on transcriptome‐wide gene expression for both symbiotic and aposymbiotic anemones, but we did not observe strong interactions between symbiotic state and *Vibrio* exposure. There were 4,164 significantly differentially expressed (DE) genes for *Vibrio* exposure, 1,114 DE genes for aposymbiosis, and 472 DE genes for the additive combinations of *Vibrio* and aposymbiosis. KEGG enrichment analyses identified 11 pathways—involved in immunity (5), transport and catabolism (4), and cell growth and death (2)—that were enriched due to both *Vibrio* and/or aposymbiosis. Immune pathways showing strongest differential expression included complement, coagulation, nucleotide‐binding, and oligomerization domain (NOD), and Toll for *Vibrio* exposure and coagulation and apoptosis for aposymbiosis.

## 1 INTRODUCTION

Cnidarians represent one of the earliest animal groups (Steele, David, & Technau, 2011) and thus are ideal systems to study the origins of genetic processes like innate immunity (Bosch, 2013; Hemmrich, Miller, & Bosch, 2007; Lehnert, Burriesci, & Pringle, 2012). Initial characterizations of cnidarian immune genes indicated that they possess key components of the major innate immune pathways including Toll/TLR pathway, complement C3, membrane attack complex/perforin domains, and other components of innate immunity once thought to have evolved much later (Miller et al., 2007; Nyholm & Graf, 2012; Putnam et al., 2007; Shinzato et al., 2011); yet, it was not known whether cnidarians used these immune pathways to mount a response against pathogens. A number of groups have since used RNA‐Seq data to produce some of the first profiles of anthozoan innate immunity (Anderson, Walz, Weil, Tonellato, & Smith, 2016; Burg, Prentis, Surm, & Pavasovic, 2016; Fuess, Mann, Jinks, Brinkhuis, & Mydlarz, 2018; Fuess, Pinzón, Weil, Grinshpon, & Mydlarz, 2017; Libro, Kaluziak, & Vollmer, 2013; Libro & Vollmer, 2016; Pinzón et al., 2015; Poole & Weis, 2014; Vidal‐Dupiol et al., 2011; Weiss et al., 2013). To date, at least nine studies have profiled the immune response of corals and their anthozoan relatives, and the data suggest that the immune response varies across anthozoans and/or immune exposures. For example, Weiss et al. (2013) studied the response of the reef coral *Acropora millepora* to the bacterial cell wall derivative muramyl dipeptide (MDP) and observed the up‐regulation of GTPases of immunity‐associated proteins (GiMAPs), which are primarily associated with immunity in vertebrates (Wang & Li, 2009) and plants (Liu, Wang, Zhang, & Li, 2008). Vidal‐Dupiol et al. (2011) compared the transcriptomic responses of the reef coral *Pocillopora damicornis* to thermal stress and *Vibrio coralliilyticus* infection and observed that immune pathways—including Toll/TLR, complement, prophenoloxidase, and the leukotriene cascade pathways—were up‐regulated due to *Vibrio* exposure. Libro et al. (2013) compared the immune response of healthy and White Band Disease (WBD) infected *Acropora cervicornis* coral using RNA‐Seq and found that C‐type lectins, ROS production, arachidonic acid metabolism, and allene oxide production were strongly up‐regulated in diseased corals (Libro et al., 2013). Up‐regulation of C‐type lectins and ROS production are hallmarks of phagocytosis, and the metabolism of arachidonic acid via the allene oxide pathway has been linked to eicosanoid synthesis in wounded corals (Lõhelaid, Teder, Tõldsepp, Ekins, & Samel, 2014). Interestingly, Libro et al. (2013) did not identify strong up‐regulation of genes associated with the classic innate immune pathways such as Toll‐like receptor pathway or prophenoloxidase pathway.

Reef‐building corals and other anthozoans like the symbiotic anemone *Exaiptasia* are also well known for their symbiotic relationship with the dinoflagellate *Symbiodinium* (also called zooxanthellae). This symbiosis presents a challenge with regard to the immune system because both pathogens and symbionts can elicit an allorecognition response, with the difference being that pathogens are typically eliminated, while symbionts are allowed to coexist within vacuoles in the endodermis of host cells (Kazandjian et al., 2008; Wakefield, Farmer, & Kempf, 2000) providing the anthozoan host up to 95% of its energy as translocated polysaccharides (Falkowski, Dubinsky, Muscatine, & Porter, 1984). Symbiosis requires clear communication between the host and symbiont. During the establishment of symbiosis, the anthozoan host must be able to recognize symbionts, engulf them in phagosomes, and shield these phagosomes from destruction (Davy, Allemand, & Weis, 2012). This suggests a clear link between symbioses and immunity wherein symbionts evade the immune response. Arrest of phagosomal maturation by Rab GTPases (Davy et al., 2012) and suppression of immune responses by transforming growth factor beta (TGF*β*) (Detournay, Schnitzler, Poole, & Weis, 2012) have been identified as potential mechanisms by which symbionts are shielded from destruction by the immune system. Once symbiosis is established, the host must regulate the growth of the symbionts and remove dead or dying symbionts (Davy et al., 2012). Regulation of nutrients has been identified as one mechanism by which the host can prevent overgrowth of the dinoflagellates (Davy et al., 2012).

Bleaching occurs when the symbionts are degraded or expelled by the coral host due to factors like thermal stress (Fitt, Brown, Warner, & Dunne, 2001), UV exposure (Gleason & Wellington, 1993), and disease (Libro et al., 2013). In addition to these naturally occurring stressors, chemical agents have been identified to deliberately induce bleaching in the laboratory for manipulative studies. These include menthol (Wang, Chen, Tew, Meng, & Chen, 2012) and photosynthesis inhibitors (Jones, 2004) that result in bleaching. Several mechanisms have been identified that result in the degradation and expulsion of the symbionts, including apoptosis, necrosis, and symbiont digestion via autophagy (symbiophagy; Dani et al., 2016), and the mechanisms vary depending on the type of stress. Apoptosis and necrosis predominate in heat‐stress bleaching, while symbiophagy predominates in menthol bleaching (Dani et al., 2016; Wang et al., 2012). Arrest of phagosomal maturation is required for the establishment of symbiosis, and Dani et al. (2016) suggest that a re‐engagement of phagosomal maturation is involved in the breakdown.

A number of transcriptomic studies of anthozoan bleaching have shown varied immune responses. Mansfield et al., 2017 found that NF‐κβ protein levels increase after bleaching and decrease after re‐colonization in *Exaiptasia*. Pinzón et al. (2015) found that 1 year after a bleaching event in *Orbicella faveolata* colonies, 17 immune genes within tumor necrosis factor pathway, apoptosis, cytoskeleton, transcription, signaling, and cell adhesion and recognition were down‐regulated. Seneca and Palumbi (2015) found that the transcriptome response of *Acropora hyacinthus* exposed to heat varied widely between the initial heat exposure and the bleaching response 15 hr later, and the later response included up‐regulation of immune and apoptosis pathways including Toll‐like receptor and C‐type lectins. In this study, we explore whether breakdown of symbiosis triggered by exposure to menthol alters the subsequent immune response to the coral pathogen *Vibrio coralliilyticus*.

The symbiotic anemone *Exaiptasia* has become a powerful model for studying symbiosis and immunity in symbiotic anthozoans because (a) it is a hardy animal that can be made aposymbiotic experimentally by exposing it to cold and heat stress (Lehnert et al., 2014), as well as by treating it with compounds like menthol (Matthews, Sproles, & Oakley, 2016), (b) it can be propagated clonally (Sunagawa et al., 2009), and (c) a well‐annotated genome for *Exaiptasia* now exists (Baumgarten et al., 2015). Limited gene expression data also exist for *Exaiptasia* comparing aposymbiotic and symbiotic anemones (Lehnert et al., 2014), *Exaiptasia* exposed to pathogens (Poole, Kitchen, & Weis, 2016), and *Exaiptasia* colonized by heterologous symbionts (Matthews et al., 2017). Lehnert et al. (2014) used RNA‐Seq to compare symbiotic and aposymbiotic anemones and identified 900 differentially expressed genes involved in metabolite transport, lipid metabolism, and amino acid metabolism. Poole et al. (2016) used qPCR to compare complement activity in response to colonization with *Symbiodinium* and the response to pathogen exposure (*Serratia marcescens*). Within the complement pathway, B‐factor 1 and MASP were up‐regulated and B‐factor 2b down‐regulated in response to both pathogen exposure and symbiont colonization. Matthews et al. (2017) used RNA‐Seq to profile immune and nutrient exchange activity in response to colonization with *Symbiodinium trenchii* versus its normal symbiont, *Symbiodinium minutum*. The expression pattern after colonization with the heterologous *S. trenchii* was intermediate between the aposymbiotic state and the normal (*S. minutum*) symbiotic state, with up‐regulation of innate immune pathways in response to heterologous colonization.

In this study, we explore the genetic links between the anthozoan–algal symbioses and immunity in a two‐factor RNA‐Seq experiment using both symbiotic and aposymbiotic *Exaiptasia* exposed to the bacterial pathogen *Vibrio coralliilyticus*. Menthol bleaching was used to compare symbiotic (untreated) versus aposymbiotic (menthol‐treated) anemones where the hypothesized mechanism of menthol bleaching is thought to be the activation of autophagic digestion of *Symbiodinium* cells (symbiophagy) as part of host innate immunity (Dani et al., 2016). The bacterial pathogen *Vibrio coralliilyticus* was used to initiate the immune response of *Exaiptasia* 72 hr after exposure to menthol. The two‐factor design comparing *Vibrio* and aposymbiosis as factors allowed us to identify gene expression patterns that were due to *Vibrio* and/or symbiotic state as well as any interactions between pathogen exposure and symbiotic state.

## 2 METHODS

**Figure 1:**
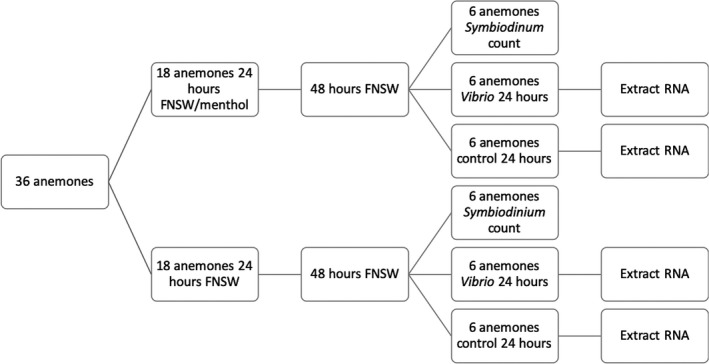
Outline of experimental design ‐ Thirty‐six anemones were divided into aposymbiotic and symbiotic. The aposymbiotic and symbiotic groups were then further subdivided with six symbiotic and six aposymbiotic anemones being sacrificed to estimate *Symbiodinium* densities due to menthol exposure leaving six symbiotic and six aposymbiotic anemones for *Vibrio* treatment and six symbiotic and six aposymbiotic controls

Wild *Exaiptasia pallida* were obtained from Carolina Biological Supply. These anemones collected off the coast of North Carolina are the source population from which the widely used cc7 clonal population was developed (Sunagawa et al., 2009). Anemones were maintained in 6‐well culture plates and held under 24‐watt t5 fluorescent lights for a 12‐hr light cycle. To avoid any bias based on lighting intensity or other positional effects, the wells of the plates were randomly assigned to six groups (Figure 1). Thirty‐six anemones were divided into aposymbiotic and symbiotic. The aposymbiotic and symbiotic groups were then further subdivided with six symbiotic and six aposymbiotic anemones being sacrificed to estimate *Symbiodinium* densities due to menthol exposure leaving six symbiotic and six aposymbiotic anemones for *Vibrio* treatment and six symbiotic and six aposymbiotic controls. At the end of the *Vibrio* exposure, we produced RNA‐Seq data for six replicate anemones for each of the four groups: *Vibrio*/symbiotic, control/symbiotic, *Vibrio*/aposymbiotic, and control/aposymbiotic.

After plating and group assignment, anemones were maintained in the wells for a 1‐week acclimation phase. They were exposed to a 12‐hr day/night cycle with a light intensity of 70 μmol quanta m^−2^ s^−1^. To avoid contamination of the RNA with any partially digested food, the anemones were not fed during the acclimation phase. Aposymbiotic anemones were produced by exposure to 0.58 mM menthol/ASW using a modified version of the protocol outlined by Wang et al. (2012). The menthol exposure was on a 72‐hr cycle, with a 24‐hr menthol exposure followed by a 48‐hr resting period in 0.2 μm filtered natural seawater (FNSW). During the menthol treatment cycle, 18 anemones had water replaced with 0.58 mM menthol/FNSW, and 18 had water replaced with fresh FNSW. The degree of menthol bleaching was measured by homogenizing anemones with BioMasher mortar and pestle sets in 1 ml FNSW, counting *Symbiodinium* cells manually with a hemocytometer from three 0.44 μl replicates per anemone, and normalizing symbiont densities by animal wet weight (after blotting to remove excess water) for six controls and five menthol‐treated anemones. Menthol‐bleached anemones had a 22.5‐fold reduction in *Symbiodinium* cells/mg wet weight (*df* = 1, *F* = 62.94, *p* = 2.37e‐05), with a control mean of 24,901 cells/mg (±7,000), and bleached mean of 1,105 cells/mg (±297).

After the menthol treatment and resting period were complete, 12 anemones were then exposed to live *Vibrio* at a concentration of 10^8^ CFU/ml in 0.2 μm filtered natural seawater (FNSW) using *Vibrio coralliilyticus* strain BAA‐450™ from ATCC^®^ (Ben‐Haim et al., 2003), and 12 controls were exposed to FNSW. The *Vibrio* inoculate was produced by centrifuging marine broth cultures for 2 min at 5,000 rcf, drawing off the broth, and re‐suspending the pellet in FNSW. The anemones were exposed to either *Vibrio* or FNSW for 24 hr and then immediately homogenized in Tri‐Reagent for downstream extraction of total RNA.

After the menthol treatment and *Vibrio* exposures were complete, the anemones were homogenized using BioMasher mortar and pestle sets for total RNA extraction. Each anemone was first homogenized in 900 μl Tri‐Reagent, and then, the 900 μl was divided into three separate tubes to which an additional 600 μl Tri‐Reagent was added to ensure sufficient Tri‐Reagent volume to lyse the cells completely. Total RNA was isolated using the Tri‐Reagent manufacturer's protocol. Total RNA was quantified on an Agilent BioAnalyzer to obtain concentrations and RNA integrity number (RIN) scores. For each of the 24 samples, the RNA isolate with the highest RIN score (mean score 6.88) was selected to proceed to mRNA isolation and Illumina RNA‐Seq library preparation. mRNA was isolated using the NEBNext® Poly(A) mRNA Magnetic Isolation Module and Illumina libraries were produced using the NEBNext® Ultra™ Directional RNA Library Prep Kit for Illumina®. Multiplexed 100‐bp paired‐end libraries were sequenced on an Illumina HiSeq 2500 platform at the FAS Center for System Biology at Harvard University. The reads were adapter and quality‐trimmed using Trimmomatic version 0.36 (Bolger, Lohse, & Usadel, 2014) using a 4‐base sliding‐window quality cutoff of 30 (Phred + 33) and the TruSeq3 adapter sequence file (TruSeq3‐PE.fa). Transcript counts were quantified against the predicted coding sequences using Salmon (Patro, Duggal, Love, Irizarry, & Kingsford, 2017), and the transcript counts were imported into DESeq2 (Love, Huber, & Anders, 2014) using tximport (Soneson, Love, & Robinson, 2015). We performed two‐way ANOVA on the counts of trimmed and aligned reads and found no significant differences based on *Vibrio* (*F* = 1.022, *p* = 0.324) or symbiotic state (*F* = 0.571, *p* = 0.459).

To facilitate KEGG (Kanehisa, Sato, Kawashima, Furumichi, & Tanabe, 2016) pathway analysis, the transcripts were mapped from *Exaiptasia* predicted protein IDs to KEGG ortholog IDs. Predicted coding sequences were extracted from the *Exaiptasia* genome annotation file using the gffread utility from the Cufflinks (Trapnell et al., 2012) package yielding 26,042 sequences. The FASTA file produced by gffread was aligned to Swiss‐Prot (The Uniprot Consortium, 2017) using blastx (Camacho et al., 2009). Swiss‐Prot hits were filtered using an e‐value cutoff of 1e^−10^ and a minimum query coverage of 50%. Where blastx hits mapped to multiple KEGG orthologs, the ortholog with the query coverage closest to 100% was selected. For multiple hits with identical coverage, the lowest e‐value was chosen. This same process was then applied in the reverse direction to eliminate duplicate *Exaiptasia* to KEGG ortholog mappings. The filtering and mapping were accomplished with custom Perl and R scripts, yielding 4,807 one‐to‐one *Exaiptasia* to KEGG ortholog mappings. This mapping table is available in the Dryad repository.aipAnnot.csv.

PERMANOVA and MDS analyses were used to identify transcriptome‐wide differences in gene expression due to symbiotic state or *Vibrio* exposure. Hellinger‐transformed DESeq2‐normalized counts were analyzed using PERMANOVA to identify transcriptome‐wide differences in expression patterns using the adonis function within the R package Vegan (Oksanen et al., 2018) with 999 permutations and formula *Vibrio* * Symbiotic State. MDS was used to visualize transcriptome‐wide differences between groups (Oksanen et al., 2018). A two‐factor negative binomial GLM implemented in DESeq2 (Love et al., 2014) was used to identify differentially expressed (DE) genes that differed due to *Vibrio* and aposymbiosis as well as the interaction. The R package ESGEA (Alhamdoosh et al., 2017) was used to identify KEGG pathways that showed significant enrichment due to *Vibrio* or aposymbiosis, and the R package GOseq (Young, Wakefield, Smyth, & Oshlack, 2010) was used to identify pathways overrepresented in DE genes.

## 3 RESULTS

RNA‐Seq data were produced for 24 anemones with six replicates each for the four treatment groups: (a) *Vibrio*/symbiotic, (b) control/symbiotic, (c) *Vibrio*/aposymbiotic, and (d) control/aposymbiotic. Menthol treatment resulted in a significant 22‐fold reduction in *Symbiodinium* densities (ANOVA *df* = 1, *F* = 18.13, *p* = 0.002); none of the anemones died during the treatment and aposymbiotic (menthol‐treated) anemones appeared completely white in color. Aposymbiotic anemones had *Symbiodinium* densities averaging 1,105 cells/mg (±297) compared to the untreated symbiotic anemones, which averaged 24,901 cells/mg (±7,000). None of the anemones died after *Vibrio* exposure, and there were no visible differences in appearance or behavior of the anemones between the *Vibrio* and control treatment groups. The number of mapped RNA‐seq reads per anemone averaged 7,681,952 read pairs (±671,717 SE) with no significant mapping differences due to either the symbiotic state (ANOVA *df* = 1, *F* = 0.571, *p* = 0.459) or *Vibrio* treatment (ANOVA *df* = 1, *F* = 1.022, *p* = 0.324).

### 3.1 Multivariate analyses

**Figure 2:**
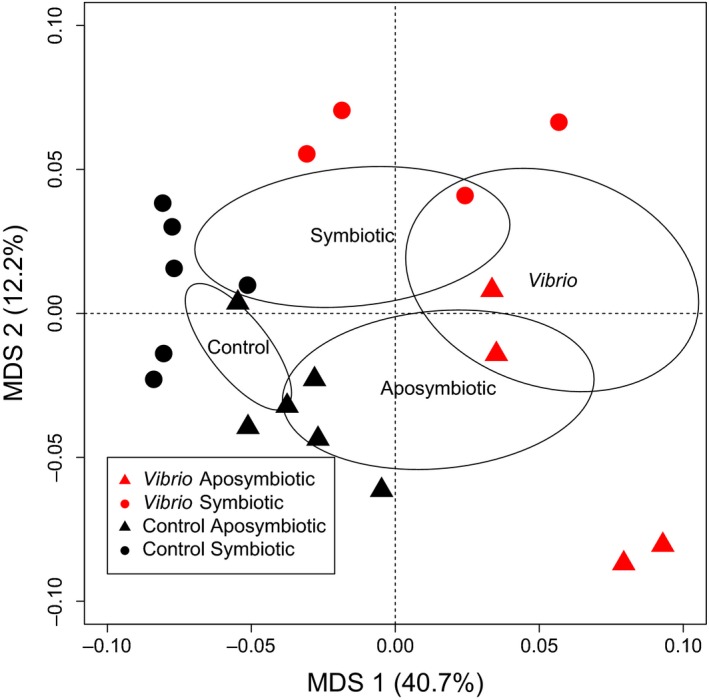
Multidimensional scaling (MDS) plot of mRNA shows differences among treatments with *Vibrio* exposure separated primarily on axis 1 and aposymbiosis separated along axis 2

Multivariate PERMANOVAs identified strong and significant differences in transcriptome‐wide gene expression patterns (Table 1) due to *Vibrio* (*r* ^2^ = 0.199, *F* = 6.07, *p* = 0.001) and aposymbiosis (*r* ^2^ = 0.098, *F* = 3.00, *p* = 0.007), but not the *Vibrio*‐aposymbiosis interaction (*r* ^2^ = 0.047, *F* = 1.432, *p* = 0.136). *Vibrio* explained 19.9% of the variation and menthol explained 9.8% of the variation. Multidimensional scaling (MDS) plots (Figure 2) show these strong differences among treatments with *Vibrio* exposure separated primarily on axis 1 and symbiotic state separated along axis 2.

**Table 1:** Multivariate PERMANOVAs show significant differences in transcriptome‐wide gene expression patterns due to *Vibrio* exposure (*p* = 0.001) and aposymbiosis (*p* = 0.007), but not the *Vibrio*‐aposymbiosis interaction

	*df*	SumsOfSqs	MeanSqs	*F*. Model	*R* ^2^	*p*‐value
Vibrio	1	0.045	0.045	6.065	0.199	0.001
Apos.	1	0.022	0.022	2.999	0.098	0.007
Vibrio: Apos.	1	0.011	0.011	1.432	0.047	0.136
Residuals	20	0.147	0.007		0.656	
Total	23	0.224			1.000	

Note

*Vibrio* exposure explained 19.9% of the variation and menthol explained 9.8% of the variation.

### 3.2 Univariate analyses

Univariate, negative binomial GLMs in DESeq2 (Love et al., 2014) were used to identify genes that were differentially expressed due to *Vibrio* exposure, aposymbiosis, and/or the interaction. In all, 4,164 genes were significantly DE for *Vibrio* exposure, 1,114 genes were DE for aposymbiosis, and 66 genes were DE for the *Vibrio*‐aposymbiosis interaction. When you consider only genes with strong protein annotations (Blast 50% query coverage, e‐value 1e^−10^, best 1:1 ortholog match), there were 1,338 DE annotated genes for *Vibrio* exposure, 462 DE annotated genes for aposymbiosis, and 11 DE annotated genes for the *Vibrio*‐aposymbiosis interaction.

**Figure 3:**
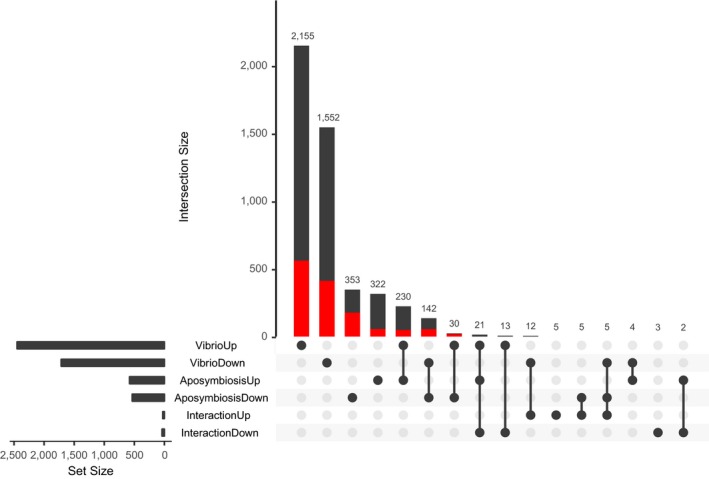
Plot of differentially expressed genes shows 2,155 genes up‐regulated and 1,552 down‐regulated for *Vibrio*, 353 genes down‐regulated and 322 up‐regulated for aposymbiosis, 230 genes up‐regulated for both *Vibrio* and aposymbiosis (V+A+), 142 genes down‐regulated for both *Vibrio* and aposymbiosis (V−A−), and 30 genes up‐regulated for *Vibrio*, but down‐regulated for aposymbiosis. Remaining groupings consisted of 21 or fewer genes. Red‐shaded areas show differentially expressed genes with KEGG ortholog annotations. Plot generated using the R package UpSetR (Conway, Lex, & Gehlenborg, 2017)

The UpSet plot (Figure 3) shows counts of genes up‐regulated and down‐regulated for both *Vibrio* and aposymbiosis. *Vibrio* alone had more DE genes up‐regulated (V+ = 2,155) than down‐regulated (V− = 1,552). Aposymbiosis alone had similar numbers of down‐regulated (A− = 353) and up‐regulated (A+ = 322) DE genes. The third major grouping consisted of 230 genes that were up‐regulated for both *Vibrio* and aposymbiosis (V+A+), and 142 genes down‐regulated for both *Vibrio* and aposymbiosis (V−A−). Thirty genes were up‐regulated for *Vibrio*, but down‐regulated for aposymbiosis. There were 11 DE interaction genes, with all remaining groupings consisting of 21 or fewer genes. The complete list of DESeq2 results, along with KEGG annotation information and BLAST e‐values, is available as Table S2.

To facilitate the analysis of the large number of differentially expressed genes, we performed KEGG pathway enrichment analysis using ESGEA (Alhamdoosh et al., 2017). We categorized pathways by KEGG pathway class and as significantly enriched for *Vibrio* only (V), aposymbiosis only (A) or both (VA). To further narrow the focus to immune‐related KEGG pathway classes, we focused on immune system, transport and catabolism, and cell growth and death pathways. Within these pathway classes, we identified 11 pathways that were significantly enriched for *Vibrio* and/or aposymbiosis (Table 2). Seven pathways were enriched for both *Vibrio* and aposymbiosis (VA), four pathways were enriched for *Vibrio* only (V), and no pathways were enriched for aposymbiosis only.

**Table 2:** Differential gene expression summary of enriched (ESGEA) and overrepresented (GOseq) pathways, counts of DE genes (DESeq2) and total annotated genes within each pathway, and DE gene counts by direction and treatment group (DE for *Vibrio*‐only up [V+] or down [V−], aposymbiosis‐only up [A+] or down [A−], Both [V+A+, V−A−, V+A−])

Pathway		Enriched		Overrepresented		Differential expression							
ID	Pathway	V	A	V	A	DE/Total	V+	V−	A+	A−	V+A+	V−A−	V+A−
Immune system													
ko04610	Complement and coagulation cascades	***	*	0.004	0.153	11/14	6	0	0	0	4	1	0
ko04062	Chemokine signaling	***	*			16/35	10	4	0	1	0	1	0
ko04612	Antigen processing and presentation					6/11	3	1	0	1	0	1	0
ko04670	Leukocyte transendothelial migration	***				12/24	6	3	0	2	0	1	0
ko04621	NOD‐like receptor signaling	***				15/43	9	3	0	2	1	0	0
Transport and catabolism													
ko04144	Endocytosis	***	*			26/89	9	9	1	3	1	2	1
ko04142	Lysosome	***	*			12/62	7	0	2	2	0	1	0
ko04146	Peroxisome	***	*			20/48	0	13	3	1	1	2	0
ko04145	Phagosome	***				13/40	8	2	1	2	0	0	0
Cell growth and death													
ko04210	Apoptosis	**	*			14/38	7	4	1	2	0	0	0
ko04115	p53 signaling	***	*			5/24	2	1	2	0	0	0	0

Note

Counts for V−A+ were all zero, so the V−A+ column was excluded. Enrichment FDR‐adjusted *p*‐values: *0.05, **0.01, ***0.005.

Differential gene expression patterns shown in Table 2 for all eleven enriched pathways indicate the numbers of annotated genes that are DE for *Vibrio* only (up [V+] or down [V−]), aposymbiosis only [A+ or A−], or both [V+A+, V−A−]. While all 11 pathways have more than 5 DE genes overall, only complement pathway had a significant over‐representation of DE genes and only for *Vibrio* (adj‐*p* = 0.004); the adjusted *p*‐value for aposymbiosis was nearly significant with an adj‐*p* equal to 0.153.

When we compare expression patterns of DE genes that are highly differentially expressed (|log_2_ fold change| > 1) across all 11 pathways, 42 genes were highly expressed (Tables 3, 4, 5). For the immune pathways (Table 3), complement and coagulation cascade pathway had the most highly expressed DE genes with 10 DE genes; four genes are part of the coagulation cascade and the remaining six genes are part of the complement cascade. Out of the four coagulation genes, two were highly up‐regulated for both *Vibrio* and aposymbiosis (TFPI1, VWF), A2MG was up‐regulated for *Vibrio* and near (|log_2_ fold change| > 1) for aposymbiosis, while F13B was up‐regulated for *Vibrio* only. Out of the six complement genes, five genes within the complement alternative pathway were up‐regulated for *Vibrio* only (MCP, DAF, CFAB, CO3, CFAH). The remaining complement gene PAI2 was down‐regulated for both *Vibrio* and aposymbiosis and is involved in negative regulation of apoptosis, fibrinolysis, and wound healing.

**Table 3:** Highly DE Immune genes by KEGG category and pathway

Gene	Symbol	Pathway	Group	*Vibrio*	Apos.
Complement and coagulation cascades					
K03909	TFPI1	Tissue factor pathway inhibitor (coagulation)	V+A+	2.03	1.94
K03900	VWF	von Willebrand factor (coagulation)	V+A+	1.42	1.44
K03906	F13B	Coagulation factor XIII B polypeptide (coagulation)	V+	2.13	0.59
K03910	A2MG	Alpha‐2‐macroglobulin (coagulation)	V+	1.86	0.93
K04007	MCP	Membrane cofactor protein (complement)	V+	6.64	0.74
K04006	DAF	Decay‐accelerating factor (complement)	V+	2.96	1.17
K01335	CFAB	Component factor B [EC:3.4.21.47] (complement)	V+	1.78	0.46
K03990	CO3	Complement component 3 (complement)	V+	1.59	0.62
K04004	CFAH	Complement factor H (complement)	V+	1.42	0.87
K19821	PAI2	Plasminogen activator inhibitor 2 (complement)	V−A−	−1.31	−1.05
NOD‐like receptor signaling					
K04570	B2CL1	Bcl‐2‐like 1 (apoptosis regulator Bcl‐X)	V+	1.96	0.55
K03174	TRAF3	TNF receptor‐associated factor 3	V+	1.79	0.22
K04729	MY88A	Myeloid differentiation primary response protein MyD88	V+	1.04	−0.17
K03173	TRAF2	TNF receptor‐associated factor 2 [EC:2.3.2.27]	V−	−1.02	−0.42
K08846	RIPK2	receptor‐interacting serine/threonine protein kinase 2 [EC:2.7.11.1]	V−	−1.36	−0.63
K04612	CASR	Calcium‐sensing receptor	V−	−1.95	−0.68
Chemokine signaling					
K11224	STAT1	Signal transducer and activator of transcription 5B	V+	2.03	0.31
K04189	CXCR4	C‐X‐C chemokine receptor type 4	V−	−1.01	−0.15
K04538	GBB4	Guanine nucleotide‐binding protein subunit beta‐4	V−	−1.02	−0.5
Antigen processing and presentation					
K06697	PSME2	Proteasome activator subunit 2 (PA28 beta)	V+	1.09	−0.59
K08062	RFXK	Regulatory factor X‐associated ankyrin‐containing protein	A−	−0.95	−1.22

Note

Within pathways genes are grouped into up‐regulated for *Vibrio* and aposymbiosis (V+A+), up‐regulated for *Vibrio*‐only (V+), down‐regulated *Vibrio* and aposymbiosis (V−A−), down‐regulated for *Vibrio*‐only (V−), down‐regulated aposymbiosis‐only (A−). The *Vibrio* and Apos. columns show log fold change.

**Table 4:** Highly DE transport and catabolism genes

Gene	Symbol	Pathway	Group	*Vibrio*	Apos.
Endocytosis					
K12182	HRS	hepatocyte growth factor‐regulated tyrosine kinase substrate	V+	1.69	0.15
K04705	HSE1	signal transducing adaptor molecule	V+	1.07	0.19
K04646	CLH	clathrin heavy chain	V+	1.04	0.4
K04189	CXCR4	C‐X‐C chemokine receptor type 4	V−	−1.01	−0.15
K12196	VPS4	vacuolar protein‐sorting‐associated protein 4	V−	−1.02	−0.22
K01115	PLD2	phospholipase D1/2 [EC:3.1.4.4]	V−	−1.31	−0.65
K13649	JUNO	folate receptor	A−	−0.3	−1.30
Peroxisome					
K00308	PAOX	N1‐acetylpolyamine oxidase [EC:1.5.3.13]	V+A+	1.57	2.00
K00306	SOX	sarcosine oxidase/ L‐pipecolate oxidase [EC:1.5.3.1 1.5.3.7]	V−	−1.04	−0.87
K01897	LCFB	long‐chain acyl‐CoA synthetase [EC:6.2.1.3]	V−	−1.33	−0.34
K11147	DHRS4	dehydrogenase/reductase SDR family member 4 [EC:1.1.‐.‐]	V−	−1.81	−0.67
K00649	GNPAT	glyceronephosphate O‐acyltransferase [EC:2.3.1.42]	V−A−	−1.48	−1.44
K03781	EASC	catalase [EC:1.11.1.6]	A+	−0.12	1.15
K00659	BAAT	bile acid‐CoA: amino acid *N*‐acyltransferase [EC:2.3.1.65 3.1.2.2]	A−	−0.89	−1.26
Lysosome					
K12396	AP3D	AP−3 complex subunit delta	V+	1.06	0.37
K04646	CLH	clathrin heavy chain	V+	1.04	0.4
K01132	GALNS	*N*‐acetylgalactosamine−6‐sulfatase [EC:3.1.6.4]	V−	−1.3	−0.7
K06129	PAG15	lysophospholipase III [EC:3.1.1.5]	A+	0.07	1.54
K01195	BGLR	beta‐glucuronidase [EC:3.2.1.31]	A−	−0.48	−1.22

**Table 5:** Highly DE cell growth and death genes

Gene	Symbol	Pathway	Group	*Vibrio*	Apos.
Apoptosis					
K04570	B2CL1	Bcl‐2‐like 1 (apoptosis regulator Bcl‐X)	V+	1.96	0.55
K04399	CASP9	caspase 9 [EC:3.4.22.62]	V+	1.65	−0.1
K04397	CASP7	caspase 7 [EC:3.4.22.60]	V+	1.4	0.69
K03173	TRAF2	TNF receptor‐associated factor 2 [EC:2.3.2.27]	V−	−1.02	−0.42
K07374	TBA	tubulin alpha	V−	−1.83	0.19
K04451	P53	tumor protein p53	A+	0.93	1.52
K08731	BIRC5	baculoviral IAP repeat‐containing protein 5	A−	−0.33	−1.01

NOD‐like receptor pathway, which includes Toll‐like receptor pathway, had six highly expressed DE genes all of which were DE for *Vibrio* only [three up‐regulated and three down‐regulated]. B2CL1, TRAF3, and MY88A were up‐regulated, while TRAF2, RIPK2, and CASR were down‐regulated due to *Vibrio* exposure.

Chemokine signaling pathway had three highly expressed DE genes for *Vibrio* only [one up; two down]. STAT1 was up‐regulated, while CXCR4 and GBB4 were down‐regulated.

Antigen processing and presentation had two highly expressed DE genes; PSME2 was up for *Vibrio* only and RFXK down for aposymbiosis only.

For the transport and catabolism pathways (Table 4), the endocytosis pathway had seven highly expressed DE genes. Three genes were up for *Vibrio* only (HRS, HSE1, CLH), three genes were down for *Vibrio* only (CXCR4, VPS4, PLD2), and one gene was down for aposymbiosis only (JUNO). Peroxisome pathway had seven highly expressed DE genes. One gene was up for both (PAOX), three genes were down for *Vibrio* only (SOX, LCFB, DHRS4), one gene was down for both (GNPAT), one gene was up for aposymbiosis only (EASC), and one gene was down for aposymbiosis only (BAAT). Lysosome pathway had 5 highly expressed DE genes. Two genes were up for *Vibrio* only (AP3D, CLH), one gene was down for *Vibrio* only (GALNS), one gene was up for aposymbiosis only (PAG15), and one gene was down for aposymbiosis only (BGLR).

For cell growth and death (Table 5), apoptosis pathway had seven highly expressed DE genes. Three genes—B2CL1 and two caspases (CASP7 and CASP9)—were up for *Vibrio* only, two genes were down for *Vibrio* only (TRAF2, TBA), one was up for aposymbiosis (P53), and one was down for aposymbiosis only (BIRC5).

## 4 DISCUSSION

Multivariate and univariate analyses of *Exaiptasia* gene expression demonstrated that exposure to live *Vibrio coralliilyticus* had strong and significant impacts on transcriptome‐wide gene expression for both symbiotic and aposymbiotic anemones, but interestingly, we did not see significant interactions between pathogen exposure and symbiotic state. In all, there were 4,164 DE genes for *Vibrio,* 1,114 DE genes for aposymbiosis, and 472 DE genes for the additive combinations of *Vibrio* and aposymbiosis. KEGG enrichment analyses identified 11 pathways—involved in immunity (5), transport and catabolism (4), and cell growth and death (2)—that were enriched due to *Vibrio* and/or aposymbiosis. Seven pathways were enriched for both *Vibrio* and aposymbiosis (complement and coagulation cascades, chemokine signaling, endocytosis, lysosome, peroxisome, apoptosis, P53 signaling), indicating overlapping genetic responses between pathogen infection and aposymbiosis. Four gene pathways were enriched for *Vibrio* alone (antigen processing and presentation, leukocyte transendothelial migration, NOD‐like receptor signaling, phagosome), demonstrating independent genetic responses underlying pathogen infection. Yet, over‐representation of DE genes was only significant for *Vibrio* exposure for complement and coagulation cascade pathway. Pathway level responses in gene expression are discussed further below.

### 4.1 Immune system response

Among the immune pathways, there was strong evidence that the complement and coagulation cascade was responding to both *Vibrio* and aposymbiosis, whereas expression of NOD/TLR pathway, chemokine, and antigen processing was initiated primarily by *Vibrio* exposure. The stimulation of complement and coagulation cascade pathway and NOD/TLR pathway indicates that bacterial immune challenge by *Vibrio* involves two of the three primary innate immune pathways in invertebrates; we did not find significant evidence for stimulation of the prophenoloxidase (PPO) activating system (i.e., melanization). The absence of a transcriptomic PPO response was surprising, because enzymatic assays of *Vibrio*‐infected *Exaiptasia* (10^6^ cfu/ml) showed a tenfold increase in PPO enzymatic activity relative to controls (Zaragoza et al., 2014), and PPO has been shown to be up‐regulated in some hard and soft coral immune responses (Palmer & Traylor‐Knowles, 2012). Differences in immune responses would be expected between *Exaiptasia* and other symbiotic anthozoans, but the conserved immune features identified in the Exaiptasia genome (Baumgarten et al., 2015) support *Exaiptasia* as a model for anthozoan immune responses.

### 4.2 Complement and coagulation cascade

Patterns of gene expression in the complement and coagulation cascade indicate that coagulation is initiated by *Vibrio* and aposymbiosis, whereas the complement alternative pathway is initiated primarily by *Vibrio* exposure. Out of the four highly expressed DE coagulation genes that differed due to *Vibrio* exposure and aposymbiosis, three genes (VFW, A2MG, TFPL1) have previously been associated with immune challenge in anemones (Rodriguez‐Lanetty, Phillips, & Weis, 2006; Stewart, Pavasovic, Hock, & Prentis, 2017) and corals (Libro et al., 2013; Libro & Vollmer, 2016; Oren et al., 2010). Von Willebrand factors (VWF) have also been observed to be up‐regulated in symbiotic *Exaiptasia* (Rodriguez‐Lanetty et al., 2006).

Von Willebrand factor (VWF) is involved in cell adhesion and collagen binding (Ruggeri, 2007) and has been associated with allogeneic rejection, pathogen exposure, and symbiotic state in cnidarians. Oren et al. (2010) observed up‐regulation of VWF during allogeneic rejection in the coral *Stylophora pistillata*, Libro et al. (2013) observed up‐regulation of VWF in response to White Band disease infection in the coral *Acropora cervicornis*, and Rodriguez‐Lanetty et al. (2006) observed up‐regulation of VWF in symbiotic versus aposymbiotic forms of the anemone *Anthopleura elegantissima*. Our data demonstrate that VWF is up‐regulated due to aposymbiosis (menthol induced) and thus the difference between our data, and Rodriguez‐Lanetty et al. (2006) may reflect differences in VWF expression between expelling zooxanthellae versus a stable aposymbiotic state. Overall, our data coupled with published data indicate that up‐regulation of VWF may be a hallmark of anthozoan immunity, allorecognition, and breakdown of symbioses.

Alpha‐2‐macroglobulin (A2MG) binds peptides including a wide range of proteinases (Borth, 1992). Its ability to bind the serine protease thrombin gives A2MG anticoagulant properties (Mitchell, Piovella, Ofosu, & Andrew, 1991), while its ability to inhibit proteins C and S gives it procoagulant properties (Cvirn et al., 2002). Many pathogen virulence factors act as proteases, and thus, A2MG's ability to inhibit proteases protects the host from these virulence factors (Armstrong & Quigley, 1999). A2MG has been associated with pathogen exposure in corals (Libro et al., 2013) and wound healing in anemones (Stewart et al., 2017), but had not been linked to aposymbioses.

Tissue factor pathway inhibitor (TFPI) inhibits coagulation by inhibition of factor Xa and factor VIIa/tissue factor (Broze & Girard, 2012) and has been associated with coral immunity (Libro & Vollmer, 2016) and oxidative stress in anemones prior to bleaching (Richier, Rodriguez‐Lanetty, Schnitzler, & Weis, 2008). Plasminogen activator inhibitor 2 (PAI2) is a serine protease inhibitor associated with inhibition of fibrinolysis (Stump, Lijnen, & Collen, 1986) and negative regulation of apoptosis (Dickinson, Bates, Ferrante, & Antalis, 1995). Down‐regulation of PAI2 for both *Vibrio* and aposymbiosis suggests a net anticoagulant effect.

Initiation of the complement cascade appeared to be stimulated only by pathogen exposure and not due to aposymbiosis. Within the complement cascade, six highly expressed DE genes were up‐regulated for *Vibrio* (C3, factor B, coagulation factor XII B polypeptide, membrane cofactor protein, decay‐accelerating factor, factor H). Complement C3 plays a central role in both the classical and alternative complement pathways. Up‐regulation of C3 has been observed in the coral *Acropora millepora* treated with bacterium *Alteromonas* (Brown, Bourne, & Rodriguez‐Lanetty, 2013) and in WBD‐infected *Acropora cervicornis* (Libro & Vollmer, 2016). Complement factor B (CFAB) is part of the alternative pathway. Poole et al. (2016) identified two variants of factor B in *Exaiptasia*, one of which was up‐regulated in response to both onset of symbiosis and treatment with *Serratia marcescens*. Up‐regulation of coagulation factor XII B chain (F13B), is associated with blood coagulation and hemostasis in vertebrates (Ivanov et al., 2017) and has also been observed in diseased *Acropora cervicornis* (Libro, 2014).

In the complement pathway, the classical and lectin pathways require specific recognition molecules for initiation, but in the alternative pathway, C3b is deposited on all cells (host as well as pathogenic) exposed to activated complement (Ferreira, Pangburn, & Cortés, 2010). Three remaining highly expressed DE genes within complement (membrane cofactor protein, complement decay‐accelerating factor, factor H) that were up‐regulated by *Vibrio* exposure are all involved in protecting host tissues from attack by the alternative complement pathway (Elvington, Liszewski, & Atkinson, 2016; Ferreira et al., 2010). Up‐regulation of membrane cofactor protein (MCP/cd46), complement decay‐accelerating factor (DAF/cd55), and component factor H in *Exaiptasia* would limit C3b deposition on healthy *Exaiptasia* cells (Elvington et al., 2016; Ferreira et al., 2010). Neither DAF, MCP, or CFAH have previously been associated with anthozoan immunity.

### 4.3 NOD/TLR pathway

*Vibrio* exposure resulted in the strong differential expression of six genes [three up and three down] in the NOD/Toll‐like receptor pathway. Myeloid differentiation primary response protein (MyD88), TNF receptor‐associated factor 3 (TRAF3), and Bcl‐2‐like 1 apoptosis regulator Bcl‐X (Bcl‐2) were up‐regulated, while TNF receptor‐associated factor 2 (TRAF2), receptor‐interacting serine/threonine protein kinase 2 (RIPK2), and calcium‐sensing receptor (CASR) were down‐regulated. MyD88, TRAF3, and RIPK2 are key regulators of the NOD and TLR pathways that lead to NF‐kappa‐β activation, cytokine secretion, and the inflammatory response (Deguine & Barton, 2014; Häcker, Tseng, & Karin, 2011; Nachbur et al., 2015), while Bcl‐2 inhibits caspases and suppresses apoptosis (Youle & Strasser, 2008). TRAF2 regulates activation of NF‐kappa‐β (Lin et al., 2011), and JNK (Brnjic, Olofsson, Havelka, & Linder, 2010) and CASR (Chakravarti, Chattopadhyay, & Brown, 2012) regulate calcium homeostasis.

Out of the six DE genes in the NOD/Toll‐like receptor pathway, only MyD88 has previously been observed to be DE in cnidarians due to immune exposure. Libro et al. (2013) observed up‐regulation of MyD88 in WBD‐infected *Acropora cervicornis*. In humans (Wang, Dziarski, Kirschning, Muzio, & Gupta, 2001), mouse (Deguine & Barton, 2014), and fly (Horng & Medzhitov, 2001), stimulation of Toll‐like receptors (TLRs) causes MyD88 to associate with the intracellular domain of the TLR leading to downstream signaling of NF‐kappa‐β via IRAK and TRAF and production of pro‐inflammatory cytokines (Akira, Uematsu, & Takeuchi, 2006). TLR activation of MyD88 has been demonstrated in the anemone *Nematostella vectensis* in a reporter gene assay where *Nematostella* TIR domain of TLR activated human MyD88. Our results indicate that MyD88 interacts with TRAF3, but not IRAK, which is supported by a MyD88 knockdown study by Franzenburg et al. (2012) in the hydrozoan *Hydra vulgaris*, which resulted in the down‐regulation of TRAF3 but not IRAK.

The expression patterns of the remaining 3 NOD/Toll‐like receptor pathway genes (Bcl‐2, RIPK2, and CASR) suggest that they are acting to prevent apoptosis in *Exaiptasia* exposed to *Vibrio*. The B‐cell lymphoma 2 (Bcl‐2) family of apoptosis‐regulating proteins includes both pro‐ and antiapoptotic members. Ainsworth et al. (2015) identified up‐regulation of the pro‐apoptotic Bcl‐2 family member Bak in *Acropora hyacinthus* tissues exhibiting white syndrome, and Pernice et al. (2011) proposed that up‐regulation of Bcl‐2 is a protective response to heat‐stress‐induced apoptotic activity in *Acropora millepora*. Down‐regulation of RIPK2 and CASR also suggests an antiapoptotic role in *Exaiptasia* exposed to *Vibrio*. RIPK2 activates NF‐kappa‐β and induces cell death (McCarthy, Ni, & Dixit, 1998). Up‐regulation of CASR leads to apoptosis in rat myocytes exposed to LPS (Wang et al., 2013). To our knowledge, we are the first to report differential expression of RIPK2 and CASR in anthozoans.

Even though *Exaiptasia* shows strong evidence for TLR pathway activation, no *Exaiptasia* TLRs met our annotation criteria (best hit, e‐value < 1e^−10^, coverage > 50%). Two *Exaiptasia* genes (KXJ18603.1, KXJ08560.1) annotated as a relaxin receptor 2 (RXFP2) and outer membrane protein OprM (OPRM) were up‐regulated for *Vibrio* and had had blast hits for a TLR with an e‐value < 1e^−10^ and coverage greater than 50%, but the TLR was not the best hit. While it is possible that these two genes represent TLRs, more data would be needed to confirm their putative functions. Toll‐like receptors (TLRs) are transmembrane proteins consisting of an extracellular leucine‐rich repeat region (LRR) involved in pathogen recognition, and an intracellular Toll–interleukin receptor (TIR) which initiates downstream activation of NF‐kappa‐β via MyD88 (Brennan et al., 2017). A single TLR has been identified in the model anemone *Nematostella vectensis*, and its activation and downstream signaling via NF‐kappa‐β have been demonstrated in response to *Vibrio coralliilyticus* (Brennan et al., 2017). We performed a Pfam domain search on the Exaiptasia predicted proteins using hmmscan and the Pfam‐A hidden Markov model Pfam‐A.hmm (Eddy, 2011) and found a number of LRR‐containing and TIR‐containing proteins up‐regulated for *Vibrio*, but none of the predicted proteins contained both domains as expected of TLRs; this is consistent with the findings of Baumgarten et al. (2015) who did not find any proteins containing both domains in the *Exaiptasia* genome. In contrast to TLRs, NOD‐like receptors (NLRs) are present in the *Exaiptasia* genome (Baumgarten et al., 2015), but as with TLRs, we observed up‐regulation of genes in the NOD pathway, but not up‐regulation of NLRs. NOD‐like receptors are intracellular pattern‐recognition proteins that when activated lead to activation of NF‐κB and MAPK, and production of inflammatory caspases (Franchi, Warner, Viani, & Nuñez, 2009).

### 4.4 Chemokine and antigen processing

Chemokine and antigen processing pathways were also activated by *Vibrio* exposure. Chemokine pathway had three highly DE genes—signal transducer and activator of transcription 1‐alpha/beta (STAT1) was up‐regulated while C‐X‐C chemokine receptor type 4 (CXCR4), and guanine nucleotide‐binding protein subunit beta‐4 (GBB4) were down‐regulated. STAT1 mediates cellular responses to interferons (IFNs), cytokines, and other growth factors (Ramana, Chatterjee‐Kishore, Nguyen, & Stark, 2000), and up‐regulation of STAT in response to bacterial exposure has been reported in a number of invertebrates including *Anopheles gambiae* (mosquito; Barillas‐Mury, Han, Seeley, & Kafatos, 1999), *Drosophila* (Buchon, Broderick, Poidevin, Pradervand, & Lemaitre, 2009), and *Fenneropenaeus chinensis* (Chinese white shrimp; Sun, Shao, Zhang, Zhao, & Wang, 2011). Sinkovics (2015) proposed a Cnidarian origin of STAT based on genomic studies on *Nematostella vectensis*, but its role in immunity had not been confirmed by expression analysis.

Two antigen processing genes were highly DE; proteasome activator subunit 2 (PSME2/PA28 beta) was up‐regulated for *Vibrio*, and regulator factor X‐associated ankyrin‐containing protein (RFXK) was down‐regulated for aposymbiosis. Proteasomes are involved in antigen processing (Michalek, Grant, Gramm, Goldberg, & Rock, 1993) and degradation of other intracellular proteins (Tanaka, 2009), including cytotoxic damaged proteins resulting from the oxidative stress of an immune response (Kammerl & Meiners, 2016). Traylor‐Knowles, Rose, Sheets, and Palumbi (2017) observed up‐regulation of proteasome components in *Acropora hyacinthus* exposed to heat stress. To our knowledge, we are the first to report up‐regulation of proteasomal proteins in response to bacterial immune challenge in cnidarians.

### 4.5 Transport and catabolism

Within transport and catabolism (peroxisome, endocytosis, lysosome), there were more genes highly down‐regulated than up‐regulated with five up‐regulated (HRS, HSE1, CLH, PAOX, and AP3D) and eight down‐regulated (CXCR4, VPS4, PLD2, SOX, LCFB, DHRS4, GNPAT, and GALNS) for *Vibrio* and three up‐regulated (PAOX, EASC, and PAG15) and four down‐regulated (JUNO, GNPAT, BAAT, and BGLR) for aposymbiosis. Once a pathogen has been recognized, endosomes, lysosomes, and peroxisomes are involved in their engulfment, destruction, and clearance (Di Cara, Sheshachalam, Braverman, Rachubinski, & Simmonds, 2017).

### 4.6 Endocytosis

Endocytosis pathway had six highly expressed DE genes; three genes were up‐regulated for *Vibrio* (HRS, HSE1, CLH), two genes were down‐regulated for *Vibrio* (VPS4, PLD2), and one gene was down‐regulated for aposymbiosis (JUNO). Following recognition by Toll‐like receptors, pathogens are engulfed by clathrin‐mediated endocytosis (Husebye et al., 2006). In *Drosophila*, endocytosis is required for activation of the Toll pathway, and endosomal proteins Mop and Hrs colocalize with the Toll receptor in endosomes (Huang, Chen, Kunes, Chang, & Maniatis, 2010). Although we observed more down‐regulation than up‐regulation of genes within the endocytosis pathway, those which were up‐regulated in response to *Vibrio* (hepatocyte growth factor‐regulated tyrosine kinase substrate HRS, clathrin heavy‐chain CLH, signal transducing adaptor molecule HSE1) are consistent with recognition by TLR pathway followed by clathrin‐mediated endocytosis.

### 4.7 Apoptosis—programmed cell death

Apoptosis pathway had five highly expressed DE genes; two caspases were up‐regulated for *Vibrio* (CASP7, CASP9), TBA was down‐regulated for *Vibrio*, P53 was up‐regulated for aposymbiosis, and BIRC5 was down‐regulated for aposymbiosis. Apoptosis has been proposed as a means of removing *Symbiodinium* during thermal bleaching (Dunn, Schnitzler, & Weis, 2007; Kvitt, Rosenfeld, & Tchernov, 2016; Pernice et al., 2011; Rodriguez‐Lanetty et al., 2006) as well as clearing pathogens in the immune response (Fuess et al., 2017; Libro et al., 2013). Caspases are key initiators of apoptosis (McIlwain, Berger, & Mak, 2013). Up‐regulation of CASP7 and CASP9 have not been previously reported in anthozoans, but up‐regulation of caspase‐3 was documented in WBD‐infected *Acropora cervicornis* (Libro & Vollmer, 2016). Tubulin alpha (TBA) was also down‐regulated due to *Vibrio* exposure in *Exaiptasia*. Down‐regulation of tubulin beta has been observed for WBD‐infected *Acropora cervicornis* as well (Libro & Vollmer, 2016).

Two DE apoptosis genes for aposymbiosis (P53, BIRC5) suggest apoptosis is a mechanism for menthol bleaching. Tumor protein P53 regulates a number of cell‐cycle functions including apoptosis, regulation of autophagy, cell‐cycle arrest, and senescence (Zilfou & Lowe, 2009). Lesser and Farrell (2004) observed up‐regulation of P53 in corals exposed to increased solar radiation, and Weis (2008) proposed activation of P53 by the reactive nitrogen species nitric oxide (NO) in thermally stressed corals as a mechanism of bleaching. The up‐regulation of P53 in aposymbiotic anemones may indicate that the mechanism of menthol‐induced bleaching is similar to the mechanisms of bleaching in thermal and solar radiation‐stressed corals. The second DE apoptosis gene for menthol BIRC5, also known as survivin, is an antiapoptotic caspase inhibitor (Li et al., 1998). The down‐regulation of BIRC5 for aposymbiotic anemones lends further support to apoptosis as a mechanism of menthol bleaching.

## 5 CONCLUSION

Exposure to live *Vibrio coralliilyticus* for both symbiotic and aposymbiotic anemones had strong and significant impacts on gene expression, but their effects were independent or additive, not interactive. The pathways most affected by *Vibrio* exposure were the complement and coagulation cascades, NOD/Toll receptor signaling, and apoptosis. Despite the absence of canonical NOD and Toll receptors in the *Exaiptasia* genome, the downstream signaling indicates involvement of NOD and Toll pathways in the anthozoan immune response. Future studies will be required to determine if *Exaiptasia* possess some functional equivalent to NOD‐like and Toll‐like receptors, and if so, how such receptors interact with downstream signaling pathways. Aposymbiosis resulted in the up‐regulation of genes within the coagulation cascade and pro‐apoptotic P53 as well as down‐regulation of antiapoptotic BIRC5, indicating that menthol‐induced bleaching may involve apoptotic mechanisms similar to those involved in thermal stress‐induced bleaching. While we did not see the interaction that we expected between symbiotic state and response to a pathogen, this study provides additional data points to better understand both bleaching and pathogen response in anthozoans.

## CONFLICT OF INTEREST

None declared.

## AUTHOR CONTRIBUTIONS

CLR and SVV conceived and designed the experiment. CLR generated and analyzed the data and wrote the manuscript. CLR and SVV edited the manuscript.

## Data Availability

The Illumina RNA‐Seq read data are available on NCBI SRA https://www.ncbi.nlm.nih.gov/sra under BioProject accession number PRJNA547971. Normalized read count data, DESeq2, and annotation files (.xlsx) are available on Dryad https://doi.org/10.5061/dryad.364j18m.
